# Integrated knowledge translation: A guide for primary care researchers

**DOI:** 10.4102/phcfm.v17i2.5168

**Published:** 2025-10-31

**Authors:** Robert Mash, Gulnaz Mohamoud, Mohamoud Merali, Nasreen Jessani

**Affiliations:** 1 Division of Family Medicine and Primary Care, Faculty of Medicine and Health Sciences, Stellenbosch University, Cape Town, South Africa; 2 Department of Family Medicine, Aga Khan University Hospital, Nairobi, Kenya; 3 Department of Counselling and Clinical Psychology, Aga Khan University Hospital, Nairobi, Kenya; 4 Institute of Development Studies, Brighton, United Kingdom; 5 Department of Global Health, Centre for Evidence Based Health Care, Stellenbosch University, Cape Town, South Africa

**Keywords:** knowledge translation, science communication, advocacy, primary care, primary health care

## Abstract

Researchers need to not only produce scientifically valid work but also consider how their new knowledge will impact society. Strategies to propel research into use for greater impact are part of the knowledge translation process. Traditionally, researchers presented their work at conferences and through scientific publications. While this remains an important strategy for engaging researchers and academics, it is insufficient to ensure impact with other stakeholders. Integrated knowledge translation (IKT) is a collaborative model of research co-production that involves a variety of stakeholders throughout the research process to enhance the relevance, timeliness and application of research findings for use in policy and practice. The key steps in IKT are outlined in this article and include stakeholder analysis, an engagement strategy and evaluation. For each stakeholder, the engagement strategy should consider the purpose of engagement, the key message, the most effective medium, the best messenger, the timing of engagement and the resources required.

## Introduction

Decisions need to be evidence-informed; however, several factors influence the decision-making process. Researchers need to understand the contextual, relational and political factors involved in decision-making and be willing to engage with the process in a mature and informed approach. Research evidence has greater value, therefore, when it is leveraged and used to drive positive societal change – whether to influence policy, practice or the public.

Research impact has traditionally been measured through academic metrics, including journal publications and citation statistics. Over time, researchers, practitioners, policymakers and donors have acknowledged that research needs to be reframed as an investment. The social return on investment, which extends beyond knowledge creation to knowledge use, is an equally important aspect of the work.

Research expertise is often focused on methodology and scientific writing but stops short of emphasising the importance of relationships, networks and ways to engage key actors and stakeholders implicated in, for and by the research itself. Furthermore, concerns about independence, objectivity and insufficient ‘soft skills’ hinder the translation, communication and dissemination of new knowledge into policy and practice.

Knowledge translation, metascience, evidence-to-action and implementation science, although referred to with different terminology, share the same aim: to understand the science, art, process and practice of getting evidence into use.

Strategies to propel research into use for greater impact are part of the knowledge translation process. Knowing how to communicate with whom and in what way, and in which windows of opportunity, is part of the strategy. We go into the specifics of all this through a step-by-step process in the following pages.

## Traditional approaches to the dissemination of research findings

The traditional way of sharing research results involves publishing them in a peer-reviewed scientific journal and presenting them at relevant scientific conferences. This can be an effective method for engaging with other researchers and academics in your field; however, it is less effective at reaching policymakers and other stakeholders. Nonetheless, sharing research findings with academic peers requires several important decisions and expertise.

### Scientific journals

It is essential to write in a scientific style, prepare a well-structured manuscript and adhere to the author instructions provided by the journal to which you intend to submit, following its requirements. Authors should meet the international criteria for authorship and not be added arbitrarily.^1^ A standardised checklist for your study design can help you ensure that all issues have been covered, and most checklists are available from the Equator network.^2^ The CRISP (Consensus Reporting Items for Studies in Primary Care) checklist was added recently to improve the reporting of primary care research.^3^ Pay particular attention to the medical subject headings or keywords you select, as these will influence how easily people can search for your work.^4^

Choosing the right journal is a key step that influences the visibility, impact and success of your work. It involves careful consideration of several factors to ensure alignment with your research goals and audience:

- Does this journal engage with the target audience that you want to reach? For example, midwives, nurses, family doctors or public health professionals.
- How much does it cost to publish? Check if the journal offers discounts or waivers for authors from low- and middle-income countries.
- Is this journal open access so that the work is readily available to all? Subscription-based journals are often free to publish, but they can be more difficult for readers to access. However, newer or open-access journals may offer broader dissemination, and their acceptance rates are often high.^5^
- What is the word count for original research articles? The allowed word count can vary from very low (e.g., 2500 words) to unlimited. Qualitative research often requires a higher word count.
- How quickly does the journal peer review and publish submissions? Understand the journal’s peer review process, time to decision and publication speed to align with your priorities.^6^
- Does the scope and focus of your work fit well with the journal? For example, in the *African Journal of Primary Health Care and Family Medicine* (PHCFM), the work must be from or about Africa and related to family medicine or primary health care. The journal’s philosophy may also be important. For example, some journals will publish anything that is scientifically valid (e.g. PLOS One), while others are very developmental and supportive of novice researchers (e.g., PHCFM), and others only want the most significant research (e.g. *The Lancet*).
- Beware of predatory journals that flatter you and solicit work for profit and have little to no peer review. Check how the journal is indexed or if it is in a credible directory such as the Directory of Open Access Journals.^7^
- What is the impact of the journal? The impact of journals is measured by impact factors and scores calculated from the number of citations generated by the published articles.^8^ Journals with higher impact metrics are typically more selective in what they send for peer review. Small-scale, descriptive and contextualised work may need to be submitted to national or regional journals. Take note of how widely the journal is indexed and therefore included in online search tools. Some journals help authors create impact through social media. As illustrated by this list of issues, the impact factors are not the only criteria to consider.

### Scientific conferences

Researchers can use scientific conferences to communicate their findings either as an oral presentation or a poster.

The traditional printed posters are being replaced by digital posters, which are available throughout the conference to all delegates. This allows for a larger audience and can be extended after the event, making it easy to update. When designing a poster, leave your audience with a clear message.^9^ Keep the text to a minimum and balance it with graphics and images. Avoid too many colours, font sizes and types, capitals, underlining, pixelated or squashed pictures or a poster that is the wrong size for the display. Think about the structure, visual attractiveness, quality of images and graphs, and readability. Do not forget to include branding for your funders and institutions. If you have the chance to speak, ensure that you can convey your message effectively within the available time. It is crucial to practise and receive peer feedback so that you can have a significant impact in the short time you have. There are several guides and online training opportunities to help you get this right.^9,10^

Likewise, when preparing for oral presentations, you must allocate time effectively, as you may not be able to cover everything in detail.^11^ Too often, presenters run out of time before they even get to their results. You may need to select what is most important to convey and refocus on a clear message. A rule of thumb is one slide per minute, but it obviously depends on how much time you talk per slide. Avoid scripting what you want to say on the slides and then simply reading the text aloud, as people tend to stop listening to you. Balance text with graphics and images, and use short bullet points to convey your message. Again, consider the visual attractiveness, structure, quality of the images, readability and branding. Check that the technology is working and you know how to use it effectively in advance, particularly if you plan to use audio or video. Ensure you speak clearly and slowly.

## Integrated knowledge translation

Integrated knowledge translation (IKT) is a collaborative model of research co-production that involves a variety of stakeholders throughout the research process to enhance the relevance, timeliness and application of research findings for use in policy and practice.^12^ It seeks to promote equitable partnerships and a true sense of codesign and co-ownership of the research design, process and results.^13^ There is considerable overlap of this approach with that of ‘science communication, engaged scholarship, Mode 2 research, co-production and participatory research approaches’.^14^ A master’s or doctoral thesis should include an overview of the strategy for dissemination and impact, and the IKT approach can assist with this planning. Likewise, funders often require research proposals to consider dissemination and impact seriously.

Several aspects of IKT work together over time. The process is iterative and reflective in nature, including a stakeholder analysis, a strategic engagement strategy, tailored communication products and the identification of fora for disseminating these communication materials. Networking and relationship building are at the heart of success in IKT and cannot be overemphasised. The material for this section is mainly derived from a masterclass in evidence-informed decision-making at Stellenbosch University, South Africa.^15^

### Stakeholder analysis

Integrated knowledge translation begins with an analysis of your stakeholders, or the individuals who can impact or be impacted by your research. The easiest initial step is to brainstorm stakeholders by category, such as policymakers (e.g., National Department of Health), decision-makers (e.g. District Management Teams), healthcare workers and clinicians, academics and researchers, as well as other organisations concerned about the topic. The community where you conduct your research and the public are particularly important stakeholders. Many funders emphasise the importance of community engagement and involvement in the research process.

Given that you cannot influence an entire organisation or assume that every person in the organisation shares the same interests or power, the next step is to examine the categories and identify the specific individuals you need to engage with. So, in this step, think about the position of the person of interest. For example, in the Ministry of Health, the person you need to engage with is the Deputy Director of Infectious Diseases. This allows you to be more focused and strategic in your approach from the onset. Once you have identified all the positions, you can better analyse them.

**FIGURE 1:**
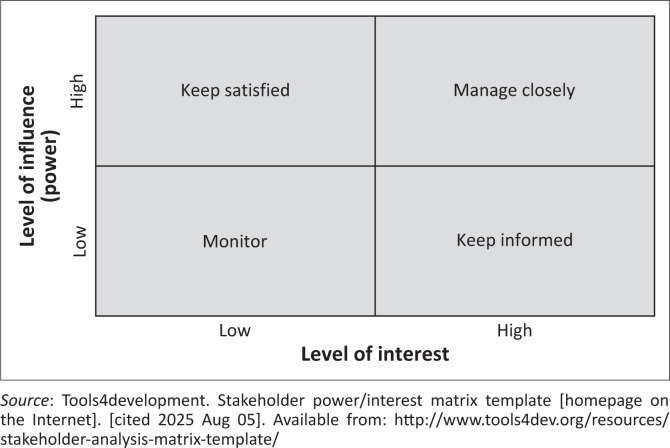
Matrix of power and interest for stakeholders.

There are many methods for stakeholder analysis. These include power versus interest matrices, network analyses and force field analyses, amongst others. In this section, we focus on one such tool, which is the power and interest matrix, as shown in Figure 1.^16^ Power refers to the level of influence the stakeholder has over the use of your research findings. For example, can they decide whether to adopt the new evidence into policy and practice? If not, are there intermediaries or brokers you need to consider? Stakeholders may also be rated in terms of their level of interest in the topic and research findings. For instance, politicians are influenced by the opinions of their voters and the public, and their interest in the issue may be linked to this.

Your level of engagement may vary depending on where you place stakeholders in this matrix:

- High influence and high interest: Engage as fully as possible and manage the relationship closely.
- High influence and low interest: Try to understand their priorities and keep them satisfied with relevant information.
- Low influence and high interest: Keep them informed about your research.
- Low influence and low interest: Monitor their level of influence and interest.

Stakeholder analysis and engagement should ideally happen throughout the process and are iterative in nature. Actors in the ecosystem often change, or their interests and power evolve. It is essential to initiate this process during the planning stage of the research study, allowing the identification of key stakeholders and, where possible, their involvement and influence on the design and conduct of the research, as well as their engagement with the findings. This should be repeated frequently during the research study as well as towards the end.

Once you have identified the right audience, you need to build relationships of trust and demonstrate how you can contribute to the broader policy mandate and priorities. It is better to argue how you can contribute to health systems and services rather than how valuable and important you or your work are.^17^ Integrated knowledge translation is much easier when stakeholders have been respected and consulted from the outset, rather than being considered only after the study is completed. Your engagement strategy will require this insight to be successful.

### Engagement strategy

For each stakeholder, a strategy can be prepared by answering the following questions.^18^

#### What is the purpose of engagement?

Clarifying the purpose of engagement links to the importance of the iterative stakeholder analysis mentioned above, as your reasons for engagement can change over time, and the person in the position may also change, which will affect your purpose and approach.

The purpose of engagement is partly addressed in the analysis above but may be further refined as ‘we need to clarify confusions on this topic’, ‘we need to compel them to action’, ‘we need them to influence person/community X’, ‘we need them to promote these results at a key meeting’ or ‘we need them to be aware of this research’.

With the public, you may also be interested in improving science literacy, building trust, correcting misinformation, or evoking advocacy and support for key recommendations.^19^ Advocacy may be needed with some stakeholders to promote a particular viewpoint or perspective. For example, in the African context, we are still actively advocating for the value of family medicine in many countries.^20^ However, it is essential to consider the potential and perils of activism, advocacy and lobbying.^21^

Researchers often view science communication as a means for expert scientists to educate the public. This may be one purpose for engagement. While it may be necessary to inform and explain your work, the nature of public engagement should be more of a dialogue to help people make sense of your work.^22^ The approach to communication can be viewed as a spectrum ranging from informing to consulting to collaborating with your audience. These are all different but important purposes. Being clear about your purpose allows for better strategic engagement.

#### What is the key message?

The first step of communicating within your IKT strategy is to define your key messages that align with your purpose. These should be clear and not ambiguous. Visualise what your pitch would be, and if it is not clear to you, it is not clear to anyone else. Remember that the magic number for our memory is three, so ensure your key message has three main points that will be remembered. How these are framed will again go back to the first two steps: Who is the audience, and what is the purpose of engagement?

The messages should be tailored to the audience in terms of their context, educational level and languages. It is, however, also helpful to explain the scientific process behind these messages and any knowledge gaps that must still be addressed through further research.

When addressing a lay audience, begin with the key messages or recommendations and their relevance to the broader context before moving on to discuss the scientific value and the underlying scientific process.^19^ This may be the opposite of a scientific presentation. It is vital to avoid scientific jargon. Stories and analogies may help a lay audience understand the findings.^23^

Advocacy with policymakers requires not only a clear understanding of your own message but also a thorough understanding of how they understand the issue.^17^ You need to be able to show how your message aligns with or links to the priorities and perspectives of your policymakers. Finding the common ground can enable your message to be heard. Be clear about your message and ensure that your constituency speaks with one voice. Avoid criticising through personalisation or politicising the issue.

#### What is the best medium and forum to convey this message?

This step combines communication as well as dissemination planning. The medium is dependent on the first three steps: Who is the audience? Why do you want to engage with them? What do you want to tell them?

Sometimes the best medium is a face-to-face or virtual meeting with a presentation or discussion. There are, of course, many options that can be tailored to your purpose and key message. For example, journal articles, social media, policy briefs, issue briefs, reviews, podcasts, opinion editorials, videos, infographics, newsletters, drama, radio or television. Publications such as The Conversation Africa allow academics to publish a summary of their work aimed at a lay audience. These articles are often republished in traditional media or lead to invitations to interviews. It is helpful to become familiar with a selection of digital or social media that you use regularly and build up a following. Infographics, pictures and graphs help summarise the information. Often several different media are needed for a single stakeholder, and may be delivered in different ways and at different time points.

Direct community or public engagement is often crucial for providing feedback to the community where the research was conducted or for facilitating a dialogue with community members. For example, in Tororo District, Uganda, the district management team and community representatives workshopped the evaluation of primary care performance and co-designed an intervention to improve primary care.^24^ Clinic health committees, hospital boards, community health forums and community advisory boards are all structures where researchers can engage with the community.

#### Who is the most effective messenger?

The researcher is not always the right person to convey the message or deliver the media, as those receiving the message need to trust the messenger. Researchers cannot be expected to possess every skill required for conducting valid science to engage stakeholders, and it may be necessary to involve other individuals. This is where we link back to the importance of networks and relationships. The message and information are more likely to be accepted if they are shared by someone considered a trusted, reliable, respected and legitimate source. Sometimes you need the help of senior faculty, journalists, broadcasters, or community leaders. Civil society groups and community-based organisations that support your viewpoint can also be helpful.

Journalists and the media are also leveraged as intermediaries, as we have seen during health emergencies. Getting your message across in the media can be a very effective method of raising awareness, engaging the public and influencing policymakers. However, engaging with the media can be daunting, as personal reputational risk is weighed against the public benefit. Preparation and media training are imperative. Researchers wishing to engage with the media are encouraged to explore such training and mentoring through readily available courses, either online or in person. Media engagement should be ethically sensitive to cultures and social norms.^25^

Public engagement can enrich your research, evoke new perspectives and questions, and enhance the relevance and trust in your work. Barriers to public engagement include a lack of time and skills, concerns about criticism from peers or potential attacks from individuals with opposing views and the challenges of engaging in dialogue with diverse groups of people. Five golden rules of effective engagement are as follows:^26^

- Acknowledge uncertainty: Science is incremental, and the ‘truth’ changes with new discoveries. Researchers should be open and transparent about the inherent uncertainties in their work.
- Avoid polarising messages: Try to avoid taking a stand at one pole of a debate and rather look for common ground and be inclusive of different views.
- Check for biases: Check for your own biases, presuppositions and assumptions when presenting your work.
- Evoke curiosity: Don’t just present your new knowledge in a dry scientific way, but look for the emotional appeal of your work, be personal and try to captivate your audience. Share stories about your work.
- Embrace complexity: Often, the topic of your work is more controversial or contested in the public space than you think. Be prepared to enter a challenging and complex dialogue.

#### When would be the best time to engage?

There may be planning and budgetary cycles that should be adhered to in terms of timing to engage. There may be moments when guidelines or policies are being reviewed or written. Sometimes, critical events in the community or society raise awareness and openness to consider your new evidence. There may be scheduled meetings or events where you can be incorporated. Your stakeholder engagement and relationships will help you identify when these are occurring and how best to leverage them. This is an equally important aspect of IKT.

#### What resources are needed to support engagement?

One of the most important steps is being able to implement your plan realistically. Suppose you do not have the human resources (e.g., necessary expertise and skill), material (e.g., design software), or financial resources (funds for travel, incentives, printing). In that case, no matter how great the plan is, you won’t be able to act on it. It is therefore crucial to consider the resources and budget required to implement the IKT strategy.

### Evaluation

Having such a clear strategy for each stakeholder enables a structured monitoring and evaluation plan.^27^ Indicators can be categorised into Reach, Usefulness, Use and Relationships. Barwick, M., et al. have an easy-to-use template available for your use.^28^

For each stakeholder, the resources needed and activities can be listed and monitored. Simple reach indicators include:

- Number of face-to-face or virtual meetings.
- Number of media articles, radio or TV slots.
- Number of social media posts and engagements.
- Number of scientific articles and citations.
- Number of conference presentations.
- Number of requests for collaboration.

Some journals now utilise services such as Altmetrics to track the impact of journal articles beyond their scientific citations, including news outlets, blogs, Wikipedia, Facebook, X, Bluesky or other platforms.^29^

Evaluation of outcomes may require a mixed-methods approach and consideration of appropriate indicators and evidence that are more qualitative in nature. These fall along the usefulness, use and relationships indicators. For example:

- Change in clinical practice seen in routine indicators or audit.
- Inclusion of research findings in curricula, policy, guidance or plans.
- Development of new relationships and collaborations.
- Change in the perspective or attitude of the stakeholder or public.

## Conclusion

Researchers need to go beyond simply disseminating their new knowledge in scientific journals and conferences, which primarily engage academics and researchers within their own field. They need to develop IKT strategies that cater to different stakeholders, tailored to their influence, impact, relationships and interests. Focusing on the intended outcome drives the force that dictates the design. Research is not an endpoint; it serves as the beginning of a journey that leads to another goal.

## References

[1] International Committee of Medical Journal Editors. Defining the role of authors and contributors [homepage on the Internet]. 2025 [cited 2025 Aug 05]. Available from: https://www.icmje.org/recommendations/browse/roles-and-responsibilities/defining-the-role-of-authors-and-contributors.html

[2] Equator Network. Enhancing the QUAlity and transparency of health research [homepage on the Internet]. 2025 [cited 2025 Aug 05]. Available from: https://www.equator-network.org/reporting-guidelines/

[3] Equator NetworkConsensus Reporting Items for Studies in Primary Care (CRISP) [homepage on the Internet]. 2025 [cited 2025 Sep 09]. Available from: https://www.equator-network.org/reporting-guidelines/improving-the-reporting-of-primary-care-research-consensus-reporting-items-for-studies-in-primary-care-the-crisp-statement/

[4] National Library of Medicine. Medical subject headings database [homepage on the Internet]. 2025 [cited 2025 Aug 05]. Available from: https://www.ncbi.nlm.nih.gov/mesh

[5] Björk B. Acceptance rates of scholarly peer-reviewed journals: A literature survey. El Prof la Inf. 2018;28(4):e280407. 10.3145/epi.2019.jul.07

[6] Ware M, Mabe M. The STM report: An overview of scientific and scholarly journal publishing [homepage on the Internet]. The Hague; 2015 [cited 2025 Oct 21]. Available from: http://digitalcommons.unl.edu/scholcom/9

[7] Directory of Open Access Journals [homepage on the Internet]. 2025 [cited 2025 Aug 05]. Available from: https://doaj.org/

[8] Dong P, Loh M, Mondry A. The “impact factor” revisited. Biomed Digit Libr. 2005;2:7. 10.1186/1742-5581-2-7 16324222 PMC1315333

[9] Brits H. PRIMAFAMED e-workshops. Presenting powerful posters [homepage on the Internet]. 2024 [cited 2025 Aug 05]. Available from: https://primafamed.sun.ac.za/2024/05/06/presenting-powerful-posters/

[10] New York University Libraries. How to create a research poster [homepage on the Internet]. New York, NY: New York University Libraries; 2025 [cited 2025 Sep 30]. Available from: https://guides.nyu.edu/posters

[11] Coetzee F, Von Pressentin K. How to prepare a presentation. In: Mash B, Brits H, Naidoo M, Ras T, editors. South African family practice manual. 4th ed. Pretoria: Van Schaik, 2023; p. 747–751.

[12] Kothari A, McCutcheon C, Graham I. Defining integrated knowledge translation and moving forward: A response to recent commentaries. Int J Health Policy Manag. 2017;6(5):299–300. 10.15171/ijhpm.2017.15 28812820 PMC5417154

[13] Graham I, Kothari A, McCutcheon C. Integrated knowledge translation research network project leads. Moving knowledge into action for more effective practice, programmes and policy: Protocols for a research programme on integrated knowledge translation. Implement Sci. 2018;13:22. 10.1186/s13012-017-0700-y 29394932 PMC5797415

[14] Nguyen T, Graham I, Mrklas K, et al. How does integrated knowledge translation (IKT) compare to other collaborative research approaches to generating and translating knowledge? Learning from experts in the field. Health Res Policy Syst. 2020;18:35. 10.1186/s12961-020-0539-6 32228692 PMC7106699

[15] Short Course Division Stellenbosch University. Evidence-informed decision making: The art, science and complexity of knowledge translation [homepage on the Internet]. [cited 2025 Aug 05]. Available from: https://shortcourses.sun.ac.za/courses/4905/

[16] Tools4development. Stakeholder power/interest matrix template [homepage on the Internet]. [cited 2025 Aug 05]. Available from: http://www.tools4dev.org/resources/stakeholder-analysis-matrix-template/

[17] Kidd M. How to be an effective advocate with your government [homepage on the Internet]. 2024 [cited 2024 Sep 29]. Available from: https://youtu.be/z26JiQpR9sc?si=XSew4AwOs_l8TUjo

[18] Bennett G, Jessani N. The knowledge translation toolkit: Bridging the know-do gap: A resource for researchers [homepage on the Internet]. Delhi: Sage; 2011 [cited 2025 Oct 21]. Available from: https://www.idrc.ca/en/book/knowledge-translation-toolkit-bridging-know-do-gap-resource-researchers

[19] Joubert M. Stellenbosch University Public Squares. From science communication to public engagement: Optimising benefits, mitigating barriers [homepage on the Internet]. 2025 [cited 2025 Aug 05]. Available from: https://www.youtube.com/watch?v=m3sQ-tPHYA4

[20] Mash R. Advocacy for family medicine in sub-Saharan Africa. Afr J Prm Health Care Fam Med. 2025;17(1):a5109. 10.4102/phcfm.v17i1.5109 PMC12421777 40922605

[21] Jessani N, Ling B, Valmeekanathan A, Babcock C, Holtgrave D. Advocacy, activism, and lobbying: How variations in interpretation affects ability for academia to engage with public policy. PLOS Glob Public Health. 2022;2(3):e0000034. 10.1371/journal.pgph.0000034 36962253 PMC10021895

[22] Hoffman A. The engaged scholar: Expanding the impact of academic research in today’s world. Stanford: Stanford University Press; 2021.

[23] Christiano A, Neimand A. The science of what makes people care. Stanford Soc Innov Rev. 2018;16(4):26–33.

[24] Besigye I, Mash R. Implementation outcomes of a community dialogue intervention to improve primary care performance in a Ugandan rural health sub-district. Glob Health Action. 2025;18(1):2541979. 10.1080/16549716.2025.2541979 40772827 PMC12333024

[25] Ward S. Handbook of global media ethics. Cham: Springer; 2021.

[26] Joubert M. The Conversation Africa. Five golden rules for effective science communication – Perspectives from a documentary maker [homepage on the Internet]. 2023 [cited 2025 Aug 05]. Available from: https://theconversation.com/five-golden-rules-for-effective-science-communication-perspectives-from-a-documentary-maker-210399

[27] Bennet A, Bennet D. Moving knowledge mobilization in the social services and the humanities: Moving from research to action. Marlinton, WV: MQI Press; 2007.

[28] Barwick M. Knowledge Translation Planning Template [homepage on the Internet]. SickKids; 2013 [cited 2025 Aug 05]. Available from: https://www.sickkids.ca/en/learning/continuing-professional-development/knowledge-translation-training/knowledge-translation-planning-template-form/

[29] Altmetric: A digital science solution [homepage on the Internet]. 2025 [cited 2025 Sep 09]. Available from: https://www.altmetric.com/about-us/

